# Cost and transportation barrier trends related to Affordable Care Act among cancer survivors and atherosclerotic cardiovascular disease patients

**DOI:** 10.1371/journal.pgph.0006791

**Published:** 2026-09-18

**Authors:** Rabia Bega, Qaidar Alizai, Charalampos M. Charalampous, Areesh Mevawalla, Meher Angez, Rida Ejaz, Lorenza Arena, Jethro Wang Zih-Shuo, Timothy M. Pawlik

**Affiliations:** Department of Surgery, The Ohio State University Wexner Medical Center and James Comprehensive Cancer Center, Columbus, Ohio, United States of America; University of Gothenburg: Goteborgs Universitet, SWEDEN

## Abstract

The Affordable Care Act (ACA) included major coverage expansions implemented in 2014. However, its association with cost‑related barriers among people with chronic disease remains incompletely described. We analyzed trends in cost and transportation related barriers to care between cancer survivors and adults with atherosclerotic cardiovascular disease (ASCVD) in relation to ACA implementation and expansion. National Health Interview Survey (NHIS) data from 2004-2024 for adults ≥18 years reporting a history of cancer or ASCVD without cancer were obtained. Primary outcomes were overall cost‑barrier and transportation barrier. We used survey weighted interrupted time series analysis (ITSA) with prespecified policy breakpoints and survey weighted modified Poisson regression to estimate associations between policy periods and barriers, adjusting for sociodemographic covariates. The analytic sample included 106,947 respondents: 63,505 cancer survivors and 43,442 adults with ASCVD without cancer. Overall cost barriers were reported by 8.1% of cancer survivors (n = 5,264) and 10.6% of adults with ASCVD without cancer (n = 4,772; p < 0.001). In adjusted models, the prevalence of overall cost barriers in 2022–2024 was lower than in 2004–2009 among both cancer survivors (aPR 0.63; 95% CI: 0.56-0.71) and adults with ASCVD without cancer (aPR 0.73; 95% CI: 0.65-0.82). In ITSA, cancer survivors had an immediate decrease in overall cost barriers at the 2022 breakpoint (-1.98 percentage points; p = 0.006). No substantial 2022 level change in overall cost barriers was observed among adults with ASCVD without cancer. Transportation barriers were higher in the post-pandemic period among years with available data, particularly among adults with ASCVD without cancer, but transportation ITSA findings were exploratory since data were unavailable in 2019–2021. Overall cost related barriers declined among U.S. cancer survivors and ASCVD patients, with the largest improvements observed in the most recent period. Nevertheless, increases in transportation-related delays underscore persistent structural barriers and highlight the need for targeted interventions to improve care accessibility.

## Introduction

Cost-related barriers to health care remain a persistent challenge in the United States, even among insured individuals [1]. Delayed care due to cost, inability to afford prescription medications, and forgone services have been associated with worse disease control, increased hospitalization, and higher mortality [2–4]. The Affordable Care Act (ACA), passed in 2009 and implemented with major coverage expansions in 2014 [5,6], aimed to improve financial protection through Medicaid expansion, insurance marketplaces, and consumer protections. Implementation of the ACA was followed by substantial gains in coverage and improvements in some access measures [1,7,8]. However, coverage does not always ensure affordability; high deductibles, cost sharing, and cumulative medical expenses can continue to deter care, particularly for lower-income adults [9]. Moreover, access to health care is multidimensional; while financial barriers are central, structural obstacles such as limited transportation can independently impede care [10]. The COVID-19 pandemic further disrupted health care utilization, insurance stability, and service delivery [11], making it important to evaluate trends in access both before and after ACA implementation, as well as the subsequent years following the pandemic [12].

Limited access is especially consequential for adults with chronic disease, for whom consistent outpatient follow-up, diagnostic testing, and medication adherence are essential to prevent disease progression and complications [2,13]. Cancer and atherosclerotic cardiovascular disease (ASCVD) are common, high-cost conditions that are susceptible to financial strain yet differ in care trajectories and utilization patterns. Cancer care often requires intensive diagnostic workups, multimodal treatment, and long-term surveillance, imposing substantial out-of-pocket and indirect costs even for insured patients [14–16]. By contrast, ASCVD typically requires sustained engagement over time; lifelong pharmacotherapy, periodic monitoring, and risk-factor modification, interspersed with episodic high-intensity treatment phases [17,18]. Given these differences, comparing long-term trends in cost-related barriers across cancer and ASCVD can help clarify how health policy reforms affect access for distinct chronic disease populations [14].

Despite major policy changes, long-term trends in cost-related barriers to care among high-risk chronic disease groups and the associated socioeconomic disparities remain incompletely characterized, particularly for cancer survivors [19,20]. Using data representative of the civilian, noninstitutionalized U.S. population, spanning more than two decades, we examined trends in self-reported cost-related barriers to care among U.S. adults with cancer compared with ASCVD as a non-cancer control. The aim of this study was to evaluate the association of ACA implementation and expansion with cost- and transportation-related barriers to care among cancer survivors versus ASCVD patients, and to assess related socioeconomic disparities.

## Materials and methods

### Data source and study design

Adults with a self-reported history of cancer or ASCVD were identified from the U.S. National Health Interview Survey (NHIS) 2004–2024 via the Integrated Public Use Microdata Series (IPUMS). NHIS is a nationally representative survey of the civilian, noninstitutionalized U.S. population and provides respondent-level data on demographics, socioeconomic characteristics, insurance coverage, and self-reported health conditions [21]. This study used publicly available, de-identified data from the National Health Interview Survey (NHIS) accessed via the Integrated Public Use Microdata Series (IPUMS). As this secondary analysis did not involve identifiable private information, it was exempt from institutional review board (IRB) approval and informed consent requirements. The data used in this study are publicly available and were accessed in February 2025; the authors did not have special access privileges. The data can be accessed through https://nhis.ipums.org/nhis/ [22]. The study was conducted in accordance with the Declaration of Helsinki (1975) and was reported following the Human Participants Research (HPR) and the cross-sectional STROBE checklist for applicable observational reporting elements only. Given the repeated cross-sectional design and interrupted time-series analysis, ITS-specific methodological details are provided in the Statistical Analyses section (**S1 Checklist**).

### Study population and exposure

Adult respondents (aged ≥18 years) were included if they answered “yes” or “no” to questions about a history of cancer or ASCVD (including coronary heart disease, angina, myocardial infarction, or stroke). Respondents with missing data for the primary cost-barrier outcome were excluded (0.71%). Missing household income-to-poverty ratio was retained as a prespecified “Unknown” category (8,326 [8.1%]). No additional respondents had missing values for the covariates included in the adjusted models; therefore, no further complete-case exclusions or missing-category recoding were required for age, sex, race/ethnicity, education, insurance, employment, or marital status.”

Individuals reporting only non‑melanoma skin cancers were also excluded [23]. Based on responses, participants were classified into two mutually exclusive groups: cancer (self-reported history of cancer) and ASCVD (self-reported ASCVD without a history of cancer). Individuals with a self-reported cancer history were assigned to the cancer cohort regardless of whether ASCVD was also reported, while the comparator ASCVD cohort was restricted to respondents with ASCVD and no cancer history. Comparing these populations permits the examination of long-term trends in cost- and transportation-related barriers differed between two clinically distinct groups with substantial chronic care needs and financial vulnerability.

### Outcomes and covariates

The primary outcome was any cost-related barrier to care, defined as self-reported delayed medical care due to cost in the past 12 months. Secondary outcomes were cost-related inability to obtain prescription medications, cost-related inability to obtain mental health care, and transportation barriers (delayed care due to unavailable or inaccessible transportation) in the prior 12 months. Age was categorized as 18–40, 41–64, and ≥65 years to reflect differences in health care utilization and the transition to Medicare eligibility at age 65 [24]. Household income was categorized using the NHIS income-to-poverty ratio as <200%, 200–400%, and ≥400% and Unknown (retained in descriptive and adjusted analyses) of the federal poverty level (FPL) [25]. All outcomes were derived from standardized NHIS questions and analyzed as binary variables. Because transportation-barrier measures were available only in 2004–2018 and 2022–2024, analyses of transportation barriers excluded 2019–2021. Transportation-barrier data were unavailable for 2019–2021 and were analyzed separately.

To assess policy-relevant changes over time, outcomes were examined within prespecified periods reflecting major health system transitions: The 2004–2009 period represented the pre-ACA baseline; 2010–2013 represented the period after ACA enactment but before major coverage expansions; 2014–2018 represented the early period after Medicaid expansion and marketplace implementation; 2019–2021 represented the late post-expansion and acute pandemic period; and 2022–2024 represented the post-pandemic/pandemic-policy transition period. Interrupted time series models used breakpoints at the start of 2009, 2014, and 2022 to estimate baseline trends, immediate level changes, and post-breakpoint slope changes in reported barriers.

### Statistical analyses

Baseline characteristics and outcome prevalence for cancer and ASCVD cohorts were summarized using survey-weighted estimates. Categorical variables were presented as frequencies with weighted proportions; between-group differences were assessed with survey-weighted Pearson χ² tests. Adjusted associations with cost-related barriers were estimated using survey-weighted modified Poisson regression with robust variance estimators to yield adjusted prevalence ratios (aPRs) and 95% confidence intervals (CIs). Separate multivariable models were fit for each outcome, with key analyses stratified by cohort (cancer vs. ASCVD). All models adjusted for prespecified covariates including survey period, age group, sex, race/ethnicity, educational attainment, insurance type, FPL, employment status, and marital status. To assess whether findings among cancer survivors were driven by respondents with comorbid ASCVD, a sensitivity analysis excluding cancer survivors with self-reported ASCVD or missing ASCVD status was conducted.

Temporal trends and changes at policy-relevant time points were evaluated using interrupted time-series analysis with segmented regression of annual survey-weighted prevalence estimates for each outcome and cohort. Models included terms for calendar time, immediate level changes at prespecified breakpoints, and post-breakpoint slope changes. Breakpoints were specified at 2009, 2014, and 2022 to correspond to the transition into the ACA implementation period, ACA coverage expansion, and the post-pandemic period, respectively. ITSA estimates were reported as absolute percentage-point changes in annual prevalence: level changes represented immediate changes at each breakpoint, and slopes represented annual percentage-point changes within each period. For cost-related outcomes, residual autocorrelation was assessed using the Durbin-Watson statistic, Breusch-Godfrey test, and residual autocorrelation plots; models were then estimated using Newey-West heteroskedasticity- and autocorrelation-consistent standard errors with a lag of 1 year. Because transportation-barrier data were unavailable in 2019–2021, transportation models were estimated using heteroskedasticity-robust standard errors and interpreted as exploratory. Overall cost barriers were specified as the primary outcome. Prescription medication, mental health, and transportation barriers were analyzed as secondary, related access outcomes. Cost-related inability to obtain prescription medications, cost-related inability to obtain mental health care, and transportation barriers were designated as secondary outcomes. Formal multiple-testing correction was not applied because these outcomes and breakpoint-specific ITSA estimates were intended as complementary descriptive analyses of correlated access measures. In addition, secondary and breakpoint-specific findings were interpreted cautiously, with emphasis on effect sizes, confidence intervals, and consistency across analyses.

Modified Poisson regression estimates adjusted prevalence ratios comparing survey periods with 2004–2009, whereas ITSA estimates absolute changes in population-level yearly prevalence at prespecified breakpoints and associated slope changes. Therefore, the magnitude of estimates from the two approaches should not be interpreted as directly interchangeable. All analyses incorporated NHIS sampling weights, strata, and primary sampling units to account for the complex survey design and to generate nationally representative estimates among NHIS respondents reporting these conditions rather than as condition-specific nationally representative prevalence estimates. Analyses used the survey domain approach for cancer and ASCVD cohorts. All tests were two-sided, results were interpreted based on effect estimates, 95% confidence intervals, exact p-values, absolute magnitude, and consistency across analyses. Analyses were conducted in Stata/SE 17.0 (StataCorp LLC, College Station, TX) and R (R 4.5.1; R Foundation for Statistical Computing, Vienna, Austria).

## Results

### Baseline characteristics

Data were extracted for 106,947 NHIS respondents, among whom 40.6% (n = 43,442) reported ASCVD without cancer and 59.4% (n = 63,505) reported a history of cancer. Overall, 48.9% (n = 48,920) were male and 79.0% (n = 82,455) were non-Hispanic White. Compared with cancer survivors, adults with ASCVD were less likely to be female (43.2%, n = 20,545 vs. 56.4%, n = 37,482; p < 0.001) and less likely to be non-Hispanic White (70.9%, n = 29,641 vs. 84.5%, n = 52,814; p < 0.001). Adults with ASCVD had greater socioeconomic disadvantage with a higher proportion having income <200% of the FPL (36.9%, n = 18,223 vs. 23.0%, n = 17,099; p < 0.001) and Medicaid coverage (16.9%, n = 8,000 vs. 8.4%, n = 5,929; p < 0.001) compared with cancer survivors **(Table 1)**. Within the cancer cohort, 12,837 of 63,505 respondents also reported ASCVD, corresponding 19.3% in survey-weighted analyses (95% CI: 18.9%-19.7%).

**Table 1:** Baseline characteristics of Cancer and atherosclerotic cardiovascular disease (ASCVD) patients.

Characteristics	Total N = 106,947	Cancer N = 63,505	ASCVD N = 43,442	p-value
**Year**				**<0.001**
2004–2009	22,746 (24.1%)	12,446 (22.8%)	10,300 (26.0%)	
2010–2013	20,187 (18.4%)	11,413 (18.0%)	8,774 (19.0%)	
2014–2018	27,404 (24.9%)	16,432 (25.3%)	10,972 (24.2%)	
2019–2021	18,319 (16.0%)	11,611 (16.6%)	6,708 (15.2%)	
2022–2024	18,291 (16.7%)	11,603 (17.3%)	6,688 (15.6%)	
**Age**				**<0.001**
18–40	5,856 (6.8%)	3,827 (7.3%)	2,029 (6.0%)	
41–64	37,257 (39.8%)	21,462 (38.9%)	15,795 (40.9%)	
≥65	63,834 (53.5%)	38,216 (53.8%)	25,618 (53.1%)	
**Sex**				**<0.001**
Male	48,920 (48.9%)	26,023 (43.6%)	22,897 (56.8%)	
Female	58,027 (51.1%)	37,482 (56.4%)	20,545 (43.2%)	
**Race/Ethnicity**				**<0.001**
Non-Hispanic White	82,455 (79.0%)	52,814 (84.5%)	29,641 (70.9%)	
Non-Hispanic Black	11,362 (9.0%)	4,638 (6.1%)	6,724 (13.1%)	
Hispanic	8,364 (7.6%)	3,700 (5.6%)	4,664 (10.4%)	
Other	4,766 (4.4%)	2,353 (3.7%)	2,413 (5.6%)	
**Education**				**<0.001**
<High school	17,585 (15.9%)	7,665 (11.7%)	9,920 (22.0%)	
High school/GED	29,566 (28.5%)	16,358 (26.5%)	13,208 (31.4%)	
> High school	59,796 (55.6%)	39,482 (61.8%)	20,314 (46.6%)	
**Insurance**				**<0.001**
Medicaid	13,929 (11.9%)	5,929 (8.4%)	8,000 (16.9%)	
Medicare	60,435 (51.8%)	36,537 (52.1%)	23,898 (51.5%)	
Private	24,726 (28.4%)	16,980 (32.5%)	7,746 (22.3%)	
Self-pay	4,712 (4.9%)	2,336 (4.1%)	2,376 (6.0%)	
Other	3,145 (3.1%)	1,723 (2.9%)	1,422 (3.4%)	
**Federal Poverty Level**				**<0.001**
<200%	35,322 (28.6%)	17,099 (23.0%)	18,223 (36.9%)	
200–399%	31,820 (30.2%)	19,569 (30.5%)	12,251 (29.7%)	
≥400%	31,479 (33.1%)	22,285 (39.0%)	9,194 (24.5%)	
Unknown	8,326 (8.1%)	4,552 (7.5%)	3,774 (8.9%)	
**Employed**				**<0.001**
No	76,042 (67.3%)	42,972 (63.8%)	33,070 (72.6%)	
Yes	30,905 (32.7%)	20,533 (36.2%)	10,372 (27.5%)	
**Married/living with partner**				**<0.001**
No	55,755 (38.4%)	31,602 (35.9%)	24,153 (42.2%)	
Yes	51,192 (61.6%)	31,903 (64.1%)	19,289 (57.8%)	

Values are presented as unweighted frequencies and survey-weighted column percentages. P-values were calculated using survey-weighted Pearson χ² tests accounting for the NHIS complex survey design, including sampling weights, strata, and primary sampling units.

### Association with cost and transportation barriers

Both cost and transportation related barriers were reported more frequently by adults with ASCVD than by cancer survivors. Overall cost barrier (delayed medical care due to cost in the prior 12 months) was reported by 10.6% (n = 4,772) of adults with ASCVD versus 8.1% (n = 5,264) of cancer survivors (p < 0.001). Cost related inability to obtain prescription medications was reported by 11.5% (n = 4,915) of adults with ASCVD versus 7.8% (n = 4,865) of cancer survivors (p < 0.001). Cost related inability to obtain mental health care was reported by 3.1% (n = 1,308) of adults with ASCVD versus 2.7% (n = 1,643) of cancer survivors (p = 0.001). Transportation barriers were reported by 5.3% (n = 2,176) of adults with ASCVD versus 3.2% (n = 1,883) of cancer survivors (p < 0.001) **(Table 2)**.

**Table 2:** Univariable comparison of cost and transport barriers among adults with cancer and atherosclerotic cardiovascular disease without cancer.

Characteristics	Total N = 106,947	Cancer N = 63,505	ASCVD N = 43,442	p-value
Cost barriers, overall	10,036 (9.1%)	5,264 (8.1%)	4,772 (10.6%)	**<0.001**
Cost barriers to prescription medications	9,780 (9.3%)	4,865 (7.8%)	4,915 (11.5%)	**<0.001**
Cost barriers to mental health	2,951 (2.9%)	1,643 (2.7%)	1,308 (3.1%)	**0.001**
Transportation barriers	4,059 (4.0%)	1,883 (3.2%)	2,176 (5.3%)	**<0.001**

Values are presented as unweighted frequencies and survey-weighted percentages. Percentages were calculated among respondents for each outcome. P-values were calculated using survey-weighted Pearson χ² tests accounting for the NHIS complex survey design, including sampling weights, strata, and primary sampling units as per database instructions.

In models adjusted for sociodemographic and socioeconomic covariates, later survey periods were independently associated with lower prevalence of overall cost barriers relative to 2004–2009 in both cohorts. Among cancer survivors, the adjusted prevalence ratio (aPR) for 2022–2024 was 0.63 (95% CI: 0.56-0.71; p < 0.001), indicating a 37% lower adjusted prevalence compared with the reference period. Among adults with ASCVD, the corresponding aPR for 2022–2024 was 0.73 (95% CI: 0.65-0.82; p < 0.001), indicating a 27% lower adjusted prevalence. Reductions were also observed in 2019–2021 for both cancer survivors (aPR 0.73; 95% CI: 0.65-0.81; p < 0.001) and adults with ASCVD (aPR 0.69; 95% CI: 0.61-0.77; p < 0.001). Smaller reductions were observed in 2010–2013 and 2014–2018, although not all estimates reached statistical significance (**Table 3**). In sensitivity analyses excluding cancer survivors with self-reported ASCVD, the 2022–2024 association remained similar among cancer survivors without ASCVD (aPR 0.66; 95% CI: 0.58-0.75; p < 0.001), supporting the robustness of the primary cancer-cohort findings (**Table C in** **S1 Appendix**). Higher income and older age were strongly associated with lower prevalence of overall cost barriers in both cohorts. Compared with adults with household income <200% of the federal poverty level, those with income ≥400% of the federal poverty level had substantially lower adjusted prevalence of cost barriers among cancer survivors (aPR 0.31; 95% CI: 0.28-0.35; p < 0.001) and adults with ASCVD (aPR 0.33; 95% CI: 0.28-0.38; p < 0.001). Similarly, compared with adults aged 18–40 years, adults aged ≥65 years had lower adjusted prevalence of cost barriers among cancer survivors (aPR 0.25; 95% CI: 0.22-0.29; p < 0.001) and adults with ASCVD (aPR 0.35; 95% CI: 0.30-0.41; p < 0.001) (**Table 3**).

**Table 3:** Survey-weighted Modified Poisson regression models estimating adjusted prevalence ratios for overall cost barrier among adults with cancer and atherosclerotic cardiovascular disease without cancer.

Characteristics	Cancer aPR (95% CI)	p-value	ASCVD aPR (95% CI)	p-value
**Year**				
2004–2009	Reference	—	Reference	—
2010–2013	0.93 (0.85–1.01)	0.073	0.96 (0.89–1.05)	0.381
2014–2018	0.91 (0.84–1.00)	**0.040**	0.92 (0.84–1.00)	**0.048**
2019–2021	0.73 (0.65–0.81)	**<0.001**	0.69 (0.61–0.77)	**<0.001**
2022–2024	0.63 (0.56–0.71)	**<0.001**	0.73 (0.65–0.82)	**<0.001**
**Age**				
18–40	Reference	—	Reference	—
41–64	1.07 (0.98–1.17)	0.112	1.22 (1.09–1.36)	**0.001**
≥65	0.25 (0.22–0.29)	**<0.001**	0.35 (0.30–0.41)	**<0.001**
**Sex**				
Male	Reference	—	Reference	—
Female	1.27 (1.18–1.36)	**<0.001**	1.16 (1.08–1.23)	**<0.001**
**Race/ethnicity**				
Non-Hispanic White	Reference	—	Reference	—
Non-Hispanic Black	0.96 (0.85–1.07)	0.438	0.94 (0.86–1.02)	0.163
Hispanic	0.93 (0.82–1.04)	0.213	0.88 (0.79–0.98)	**0.024**
Other	0.92 (0.79–1.07)	0.300	0.98 (0.84–1.13)	0.746
**Education**				
<High school	Reference	—	Reference	—
High school/GED	0.91 (0.82–1.00)	0.058	0.85 (0.78–0.93)	**0.001**
> High school	0.99 (0.90–1.09)	0.898	1.01 (0.93–1.10)	0.871
**Insurance**				
Medicaid	Reference	—	Reference	—
Medicare	1.66 (1.45–1.90)	**<0.001**	1.69 (1.50–1.91)	**<0.001**
Private	1.35 (1.18–1.55)	**<0.001**	1.34 (1.16–1.55)	**<0.001**
Self-pay	4.23 (3.79–4.73)	**<0.001**	4.05 (3.63–4.52)	**<0.001**
Other	1.32 (1.07–1.61)	**0.009**	1.20 (0.98–1.46)	0.076
**Federal poverty level**				
<200%	Reference	**—**	Reference	—
200–399%	0.70 (0.64–0.75)	**<0.001**	0.74 (0.68–0.80)	**<0.001**
≥400%	0.31 (0.28–0.35)	**<0.001**	0.33 (0.28–0.38)	**<0.001**
Unknown	0.52 (0.46–0.60)	**<0.001**	0.55 (0.47–0.63)	**<0.001**
**Employed**				
No	Reference	—	Reference	—
Yes	0.97 (0.89–1.04)	0.385	1.06 (0.98–1.15)	0.126
**Married**				
No	Reference	—	Reference	—
Yes	0.72 (0.67–0.76)	**<0.001**	0.78 (0.73–0.84)	**<0.001**

Values are adjusted prevalence ratios and 95% confidence intervals from survey-weighted modified Poisson regression models with log link. Separate models were fitted for adults with cancer and adults with ASCVD without cancer. Models adjusted for survey period, age, sex, race and ethnicity, education, insurance status, federal poverty level, employment status, and marital/partner status. The full analytic cohort included 106,947 respondents; regression models were estimated among respondents with nonmissing overall cost-barrier outcome data. P-values were calculated using survey-adjusted Wald tests.

### Interrupted time series analyses

**Fig 1:**
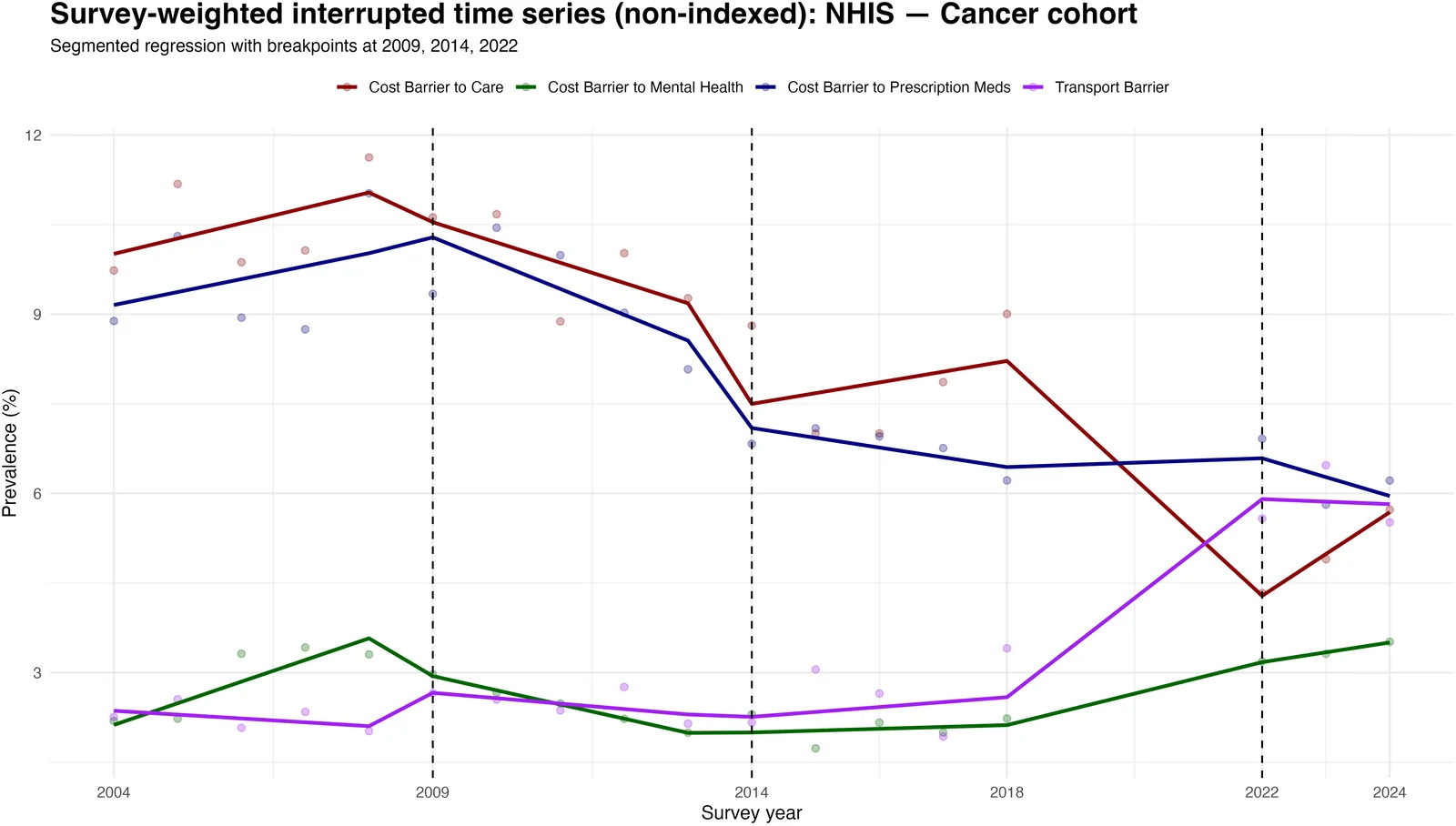
Survey-weighted Interrupted time series analysis of cost-barriers and transportation barriers to healthcare among adults with cancer.

**Fig 2:**
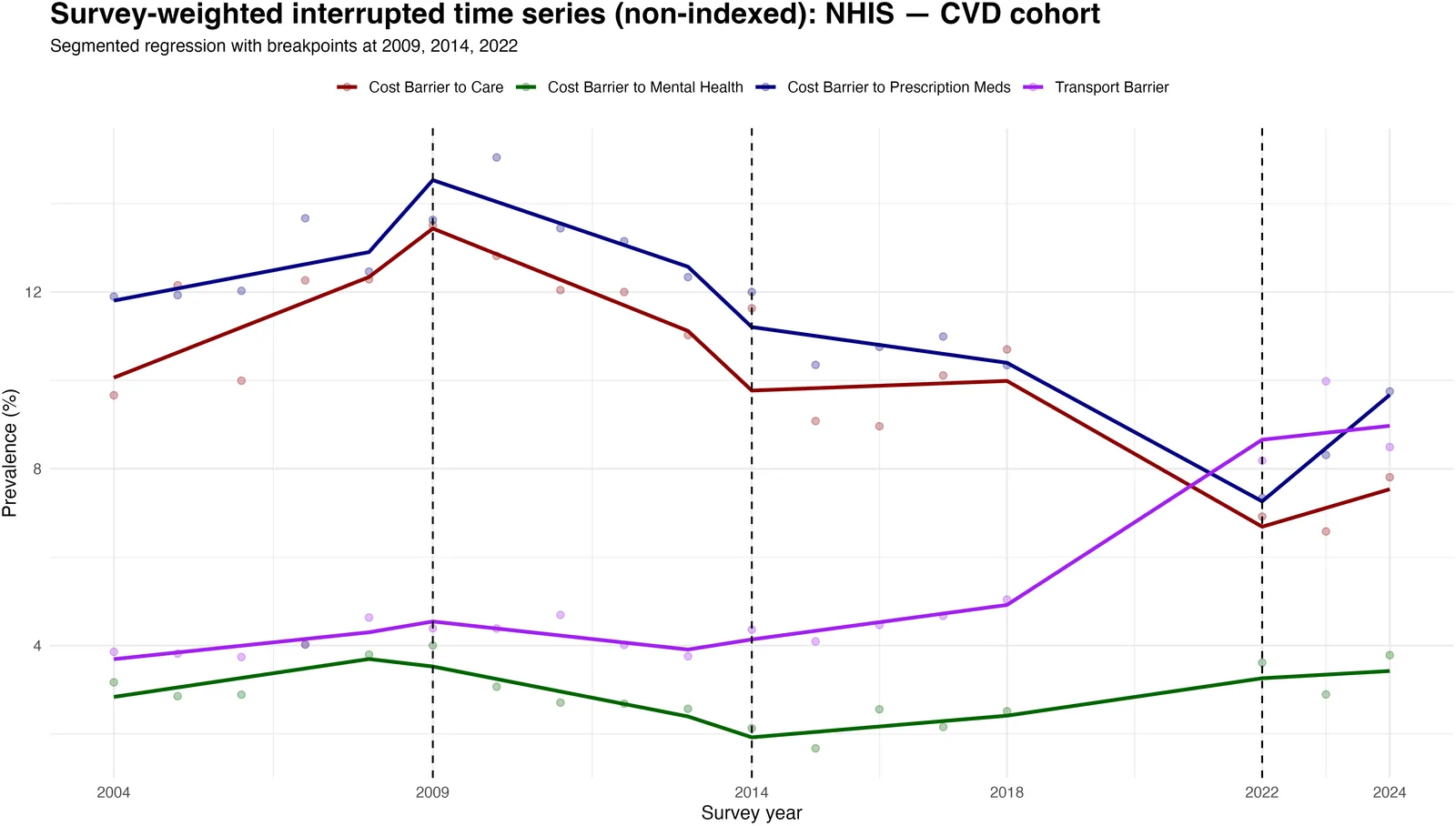
Survey-weighted Interrupted time series analysis of cost-barriers and transportation barriers to healthcare among adults with atherosclerotic cardiovascular disease.

In interrupted time-series analyses, no immediate level changes in overall cost barriers were observed at the 2009 or 2014 breakpoints among cancer survivors. At the 2022 breakpoint, cancer survivors had an immediate decrease in overall cost barriers (-1.98 percentage points; p = 0.006) (**Table A in** **S1 Appendix**; Fig 1). In contrast, adults with ASCVD without cancer had an immediate increase in overall cost barriers at 2009 (1.60 percentage points; p < 0.001), but no clear level change in 2014 or 2022 (**Table B in** **S1 Appendix**; Fig 2). Secondary and breakpoint-specific findings were interpreted cautiously. Modified Poisson regression and ITSA estimates were interpreted as complementary but distinct, with Poisson models estimating adjusted relative differences across survey periods and ITSA estimating absolute percentage-point changes in annual prevalence at prespecified breakpoints.

**Fig 3:**
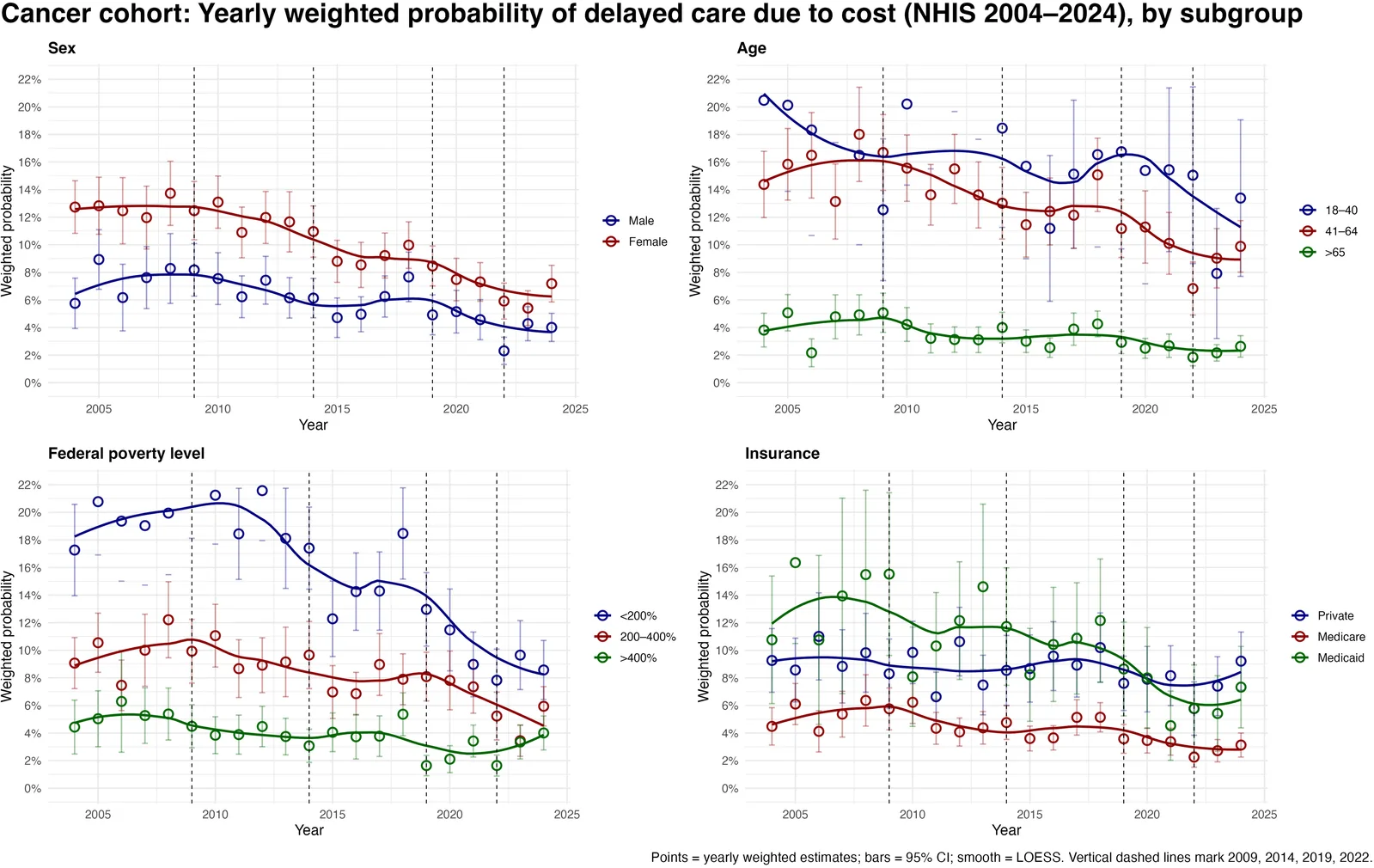
Survey-weighted trends in overall cost-barrier to care among adults with cancer stratified by sex, age group, federal poverty level, and insurance type.

Temporal trends in overall cost‑barrier varied within subgroups of both cohorts. Females consistently had higher annual weighted probabilities than males, and the absolute decline over time was larger among females **(**Fig 3**; Fig A in** **S1 Appendix**). Adults ≥65 years had the lowest and relatively stable probabilities across years, whereas younger age groups generally exhibited higher probabilities and more pronounced declines, with greater variability in the youngest stratum of the ASCVD cohort. By FPL, adults <200% experienced the largest absolute decline over time, followed by individuals at 200–400% FPL; adults ≥400% FPL remained lowest with smaller absolute changes. By insurance type, the largest downward shift occurred among Medicaid beneficiaries, while Medicare beneficiaries remained consistently lower with smaller changes. Additional stratifications by education and marital status revealed similar gradients with higher probabilities and larger absolute declines over time among adults with lower educational attainment and among individuals who were unmarried **(Figs B, Fig C in** **S1 Appendix**).

## Discussion

The current study examined two decades of NHIS data to assess long-term trends in cost-related barriers to care among U.S. adults with cancer or ASCVD. Overall, reported delays in care due to costs declined steadily across both disease groups, occurring alongside major health‑policy changes and evolving care delivery. At the same time, structural barriers most notably transportation challenges emerged as an increasing concern in the post pandemic period. Together, these findings indicate improvements in financial access for people with chronic disease, while highlighting persistent non‑financial obstacles that require attention. The study’s strengths include its large survey-weighted sample, extended time frame, and use of policy-relevant time points to examine trends in both financial and structural access barriers.

The Affordable Care Act (ACA), enacted in 2010 with major coverage expansions beginning in 2014, sought to reduce financial barriers through Medicaid expansion, subsidized marketplace coverage, and consumer protections (e.g., prohibiting denial for preexisting conditions and limiting catastrophic losses) [5]. These reforms targeted populations most vulnerable to cost barriers, particularly low‑income adults. Early evaluations documented substantial gains in coverage and access nationally [1]. Rather than producing an abrupt shift at policy milestones, the results suggest a gradual improvement in affordability‑related access, consistent with prior work demonstrating that the effects of large coverage reforms accrue over time as eligibility expands, enrollment stabilizes, and care pathways adapt [5,6,26]. The most pronounced declines in overall cost‑barriers occurred in the most recent period, which overlapped with pandemic‑era measures (e.g., Medicaid continuous enrollment and enhanced marketplace subsidies) that likely reduced insurance churn and improved short‑term affordability [1,27–29]. Although temporary, these protections operated within the broader ACA framework and may help explain why the greatest improvements were observed late in the study period underscoring the importance of sustained coverage stability, not just coverage expansion, to reduce cost‑related barriers [6,30]. These findings suggest that while ACA-related reforms improved access over time, the most pronounced reductions observed in 2022 may reflect temporary pandemic-era policy protections layered onto the ACA framework [29].

Across conditions, adults with ASCVD reported higher prevalence of overall cost‑barriers than cancer survivors, which may reflect the sustained financial demands of chronic outpatient management and long‑term medication use [7,15–17,31]. However, these between-cohort differences should be interpreted cautiously because the cancer and ASCVD cohorts differed substantially in baseline socioeconomic characteristics. Although multivariable models adjusted for these factors, residual confounding may remain; therefore, differences between cohorts should not be interpreted as reflecting disease status alone.

Stratified analyses revealed heterogeneity in trends across socioeconomic groups. While cost‑barriers declined across nearly all demographic and socioeconomic strata, the magnitude varied. Specifically, lower‑income adults experienced the largest absolute reductions consistent with insurance expansions improving financial access for individuals historically most disadvantaged whereas higher‑income groups had smaller changes, likely because they had greater baseline coverage stability [6,26,29,31–33]. By age, adults ≥65 years maintained the lowest and most stable prevalence of cost‑barriers, consistent with near‑universal Medicare coverage, while younger adults demonstrated higher baseline probabilities and steeper declines [32,34,35]. Despite reductions in financial barriers, transportation‑related barriers increased in recent years, mirroring prior work on structural impediments to care [36–38]. This divergence highlights that insurance expansion alone does not eliminate non‑financial obstacles such as transportation, geographic provider access, and system disruptions which remain important determinants of utilization [39]. The post‑pandemic rise in transportation barriers emphasizes the multidimensional nature of access and the need for complementary policies and interventions [40]. Large scale insurance reforms can yield meaningful improvements in affordability‑related access for adults with chronic disease, supporting the ACA’s intent to reduce cost‑related delays. Persistent non‑financial barriers indicate, however, that “access” must be addressed holistically. Practically, health systems should integrate access‑support strategies systematic screening for transportation and logistical barriers, streamlined referrals to care coordination and social work, benefit navigation, and patient education about covered services (including non‑emergency medical transportation where available) into routine care [41]. Although several differences across cohorts and time periods were associated with low p-values, some were modest in absolute magnitude. These findings should therefore be interpreted with attention to both statistical and practical significance. In a large, nationally representative survey, small percentage-point differences may reach statistical significance and should not be interpreted as large individual-level effects. However, even modest absolute differences may correspond to a meaningful number of adults experiencing cost- or transportation-related barriers to care at the population level.

From a research and policy perspective, monitoring both affordability and structural barriers in parallel and examining heterogeneity by geography and state policy (e.g., Medicaid expansion, local transportation infrastructure) will help target complementary investments where they are most needed [42].

The findings of the current study should be interpreted considering several limitations. Outcomes were self‑reported and subject to recall or perception bias, although self‑reported delayed care is a validated measure of access experience. The cross‑sectional NHIS design limited individual‑level causal inference; interrupted time series strengthened temporal interpretation but could not fully exclude concurrent secular influences. The broad ≥65 age category may mask heterogeneity in barriers within older populations. NHIS sampling weights are calibrated to the demographic distribution of the overall U.S. civilian, noninstitutionalized population rather than to adults with cancer or ASCVD specifically. Consequently, applying these general-population weights within disease-defined subgroups could introduce bias if subgroup distributions differ from the original calibration targets; condition-specific post-stratification anchored to reliable external benchmarks would be needed to generate truly condition-specific nationally representative estimates. Survivorship bias should be taken into consideration since NHIS excludes individuals who died, were institutionalized, or were too ill to participate, and this survivorship/selection bias may differ between cancer and ASCVD. In addition, we could not account for state level Medicaid expansion differences. Although the outcomes were prespecified hierarchically and represented correlated dimensions of access, formal correction for multiple comparisons was not applied; therefore, findings from secondary, subgroup, and breakpoint-specific analyses may include chance associations and should be considered exploratory. Cardiovascular multimorbidity within the cancer cohort may compound financial toxicity and that the mutually exclusive comparator design may obscure the specific contribution of multimorbidity. Transportation-barrier data were unavailable in 2019–2021, coinciding with the acute COVID-19 pandemic period. As a result, the 2022 transportation-barrier level-change estimates cannot determine whether observed post-pandemic differences reflected an abrupt change in 2022 or a sustained shift that began during the unobserved pandemic years. Transportation ITSA findings should therefore be interpreted as exploratory. The survey data also lacked granular clinical detail (e.g., cancer stage, time since diagnosis, ASCVD severity), and state-level data constraining assessment of disease‑severity trends and results should be interpreted as temporal associations.

## Conclusion

In this study, cost‑related barriers to care among U.S. adults with cancer and ASCVD declined substantially over the past two decades, with the greatest improvements observed in recent years overlapping pandemic era protections. These gains were most pronounced among socioeconomically vulnerable populations, suggesting that coverage expansions and stability can reduce financial barriers. However, the persistence and recent increase of transportation‑related barriers indicates that insurance coverage alone was insufficient to ensure equitable access. Future research should assess state-level cost and transportation barrier as well as address both financial and structural barriers, whereas clinicians and health systems should embed access‑support strategies into routine care for patients with chronic disease.

## Supporting information

S1 Appendix Table A. Survey-weighted Interrupted Time Series Analysis for cost and transport barrier among cancer survivors. **Table B.** Survey-weighted Interrupted Time Series Analysis for cost and transport barrier among respondents with atherosclerotic cardiovascular disease (ASCVD). **Table C.** Sensitivity analysis of adjusted associations with overall cost barriers among cancer survivors without atherosclerotic cardiovascular disease (ASCVD). **Fig A.** Survey-weighted trends in cost-barrier to care among adults with atherosclerotic cardiovascular disease stratified by sex, age group, federal poverty level, and insurance. **Fig B.** Survey-weighted trends in cost-barrier to care among adults with atherosclerotic cardiovascular disease (ASCVD) stratified by race/ethnicity, education level, employment status, and marital status. **Fig C.** Survey-weighted trends in overall cost-barrier to care among adults with cancer stratified by race/ethnicity, education level, employment status, and marital status.
(DOCX)

S1 Checklist STROBE checklist. (DOC)
