# Supplementary material for: Cost and transportation barrier trends related to Affordable Care Act among cancer survivors and atherosclerotic cardiovascular disease patients
Source: PLOS Glob Public Health. 2026 Sep 18;6(9):e0006791. doi: 10.1371/journal.pgph.0006791 (PMC13588366; doi:10.1371/journal.pgph.0006791)
Supplement: Supplementary file 1 [file pgph.0006791.s001.docx]

**S1 Appendix**

**Table A**

| **Outcome** | **2004–2008** | **p-value** | **2009–2013** | **p-value** | **2014–2021** | **p-value** | **2022–2024** | **p**  **-value** |
| --- | --- | --- | --- | --- | --- | --- | --- | --- |
| **Cost barriers, overall** | 0.267 | 0.136 | −0.336 | **0.003** | −0.249 | **0.046** | 0.698 | **<0.001** |
| **Cost barriers to prescription medications** | 0.271 | 0.258 | −0.395 | 0.098 | −0.101 | 0.355 | −0.350 | 0.145 |
| **Cost barriers to mental health** | 0.342 | **0.001** | −0.239 | **<0.001** | 0.155 | **0.010** | 0.164 | **<0.001** |
| **Transportation barriersᵃ** | −0.068 | 0.263 | −0.080 | 0.269 | 0.136ᵇ | 0.506 | −0.031 | 0.918 |
|  |  |  |  |  |  |  |  |  |
| **Outcome** | **2009** | **p-value** | **2014** | **p-value** | **2022** | **p-value** |  |  |
| **Cost barriers, overall** | −0.731 | 0.304 | −0.620 | 0.374 | −1.984 | **0.006** |  |  |
| **Cost barriers to prescription medications** | −0.230 | 0.868 | −1.076 | 0.107 | 0.356 | 0.669 |  |  |
| **Cost barriers to mental health** | −0.971 | **0.007** | 0.141 | 0.530 | 0.048 | 0.869 |  |  |
| **Transportation barriersᵃ** | 0.610 | **0.008** | 0.114 | 0.815 | 2.428 | 0.121 |  |  |

Values represent absolute percentage-point changes in annual survey-weighted prevalence among cancer survivors. Segment-specific slopes represent annual percentage-point changes within each period. Immediate level changes represent absolute percentage-point changes at the specified breakpoint relative to the projected pre-breakpoint trend.

Cost-related outcomes were modeled using segmented linear regression of annual survey-weighted prevalence estimates with Newey-West heteroskedasticity- and autocorrelation-consistent standard errors with lag 1 year.

ᵃ Transportation-barrier data were unavailable in 2019–2021, resulting in an irregular annual time series. Therefore, transportation-barrier models were estimated using heteroskedasticity-robust standard errors and interpreted as exploratory.

ᵇ For transportation barriers, the 2014–2021 slope column reflects the available 2014–2018 pre-gap segment only.

Breakpoints corresponded to 2009, 2014, and 2022. The 2009 breakpoint was used to capture the ACA implementation period, the 2014 breakpoint to capture ACA coverage expansion, and the 2022 breakpoint to capture the post-pandemic period.

**Table B**

| **Outcome** | **2004–2008** | **p-value** | **2009–2013** | **p-value** | **2014–2021** | **p-value** | **2022–2024** | **p-value** |
| --- | --- | --- | --- | --- | --- | --- | --- | --- |
| **Cost barriers, overall** | 0.472 | **0.004** | −0.687 | **<0.001** | −0.576 | **0.008** | 0.284 | 0.300 |
| **Cost barriers to prescription medications** | 0.263 | 0.136 | −0.487 | 0.130 | −0.448 | **<0.001** | 1.570 | **<0.001** |
| **Cost barriers to mental health** | 0.182 | 0.145 | −0.363 | **0.002** | 0.295 | **0.018** | −0.017 | 0.961 |
| **Transportation barriersᵃ** | 0.252 | 0.119 | −0.100 | 0.287 | 0.222ᵇ | **0.011** | −0.146 | 0.796 |
|  |  |  |  |  |  |  |  |  |
| **Outcome** | **2009** | **p-value** | **2014** | **p-value** | **2022** | **p-value** |  |  |
| **Cost barriers, overall** | 1.600 | **<0.001** | 0.702 | 0.380 | 0.548 | 0.653 |  |  |
| **Cost barriers to prescription medications** | 1.735 | 0.059 | −0.659 | 0.430 | −1.313 | **0.004** |  |  |
| **Cost barriers to mental health** | 0.155 | 0.736 | −0.393 | 0.255 | −0.467 | 0.629 |  |  |
| **Transportation barriersᵃ** | −0.420 | 0.365 | 0.234 | 0.600 | 3.548 | **0.004** |  |  |

Values represent absolute percentage-point changes in annual survey-weighted prevalence among respondents with ASCVD without cancer. Segment-specific slopes represent annual percentage-point changes within each period. Immediate level changes represent absolute percentage-point changes at the specified breakpoint relative to the projected pre-breakpoint trend.

Cost-related outcomes were modeled using segmented linear regression of annual survey-weighted prevalence estimates with Newey-West heteroskedasticity- and autocorrelation-consistent standard errors with lag 1 year.

ᵃ Transportation-barrier data were unavailable in 2019–2021, resulting in an irregular annual time series. Therefore, transportation-barrier models were estimated using heteroskedasticity-robust standard errors and interpreted as exploratory.

ᵇ For transportation barriers, the 2014–2021 slope column reflects the available 2014–2018 pre-gap segment only. Breakpoints corresponded to 2009, 2014, and 2022. The 2009 breakpoint was used to capture the ACA implementation period, the 2014 breakpoint to capture ACA coverage expansion, and the 2022 breakpoint to capture the post-pandemic period.

**Table C**

| **Characteristics** | **Cancer without ASCVD aPR (95% CI)** | **p-value** |
| --- | --- | --- |
| **Year** |  |  |
| 2004–2009 | Reference | — |
| 2010–2013 | 0.97 (0.88–1.05) | 0.431 |
| 2014–2018 | 0.94 (0.85–1.04) | 0.248 |
| 2019–2021 | 0.74 (0.66–0.84) | **<0.001** |
| 2022–2024 | 0.66 (0.58–0.75) | **<0.001** |
| **Age** |  |  |
| 18–40 | Reference | — |
| 41–64 | 1.05 (0.96–1.15) | 0.261 |
| ≥65 | 0.25 (0.21–0.29) | **<0.001** |
| **Sex** |  |  |
| Male | Reference | — |
| Female | 1.30 (1.20–1.41) | **<0.001** |
| **Race/ethnicity** |  |  |
| Non-Hispanic White | Reference | — |
| Non-Hispanic Black | 0.91 (0.80–1.04) | 0.169 |
| Hispanic | 0.96 (0.84–1.09) | 0.501 |
| Other | 0.94 (0.79–1.12) | 0.511 |
| **Education** |  |  |
| < High school | Reference | — |
| High school/GED | 0.87 (0.78–0.97) | 0**.016** |
| > High school | 0.96 (0.86–1.07) | 0.417 |
| **Insurance** |  |  |
| Medicaid | Reference | — |
| Medicare | 1.68 (1.42–1.99) | **<0.001** |
| Private | 1.37 (1.17–1.60) | **<0.001** |
| Self-pay | 4.44 (3.89–5.06) | **<0.001** |
| Other | 1.40 (1.11–1.76) | **0.004** |
| **Federal poverty level** |  |  |
| <200% | Reference | — |
| 200–399% | 0.72 (0.66–0.79) | **<0.001** |
| ≥400% | 0.33 (0.29–0.37) | **<0.001** |
| Unknown | 0.54 (0.47–0.63) | **<0.001** |
| **Employed** |  |  |
| No | Reference | — |
| Yes | 1.01 (0.93–1.10) | 0.758 |
| **Married** |  |  |
| No | Reference | — |
| Yes | 0.70 (0.65–0.75) | **<0.001** |

Sensitivity analysis excluded cancer survivors with self-reported ASCVD and cancer survivors with missing ASCVD status. The cancer group therefore includes only respondents with cancer without ASCVD. Models were otherwise adjusted using the same covariates as the primary analysis. Values are adjusted prevalence ratios and 95% confidence intervals from survey-weighted modified Poisson regression models with log link. P-values were calculated using survey-adjusted Wald tests.

**Fig A**

**Fig B**

**Fig C**
